# NRG/RTOG 0837: Randomized, phase II, double-blind, placebo-controlled trial of chemoradiation with or without cediranib in newly diagnosed glioblastoma

**DOI:** 10.1093/noajnl/vdad116

**Published:** 2023-10-11

**Authors:** Tracy T Batchelor, Minhee Won, Arnab Chakravarti, Costas G Hadjipanayis, Wenyin Shi, Lynn S Ashby, Volker W Stieber, H Ian Robins, Heidi J Gray, Alfredo Voloschin, John B Fiveash, Clifford G Robinson, UshaSree Chamarthy, Young Kwok, Terrence P Cescon, Anand K Sharma, Rekha Chaudhary, Mei-Yin Polley, Minesh P Mehta

**Affiliations:** Department of Neurology, Brigham and Women’s Hospital, Boston, Massachusetts, USA; Department of Statistics, NRG Oncology Statistics and Data Management Center, Philadelphia, Pennsylvania, USA; Department of Radiation Oncology, Wexner Medical Center, Ohio State University Comprehensive Cancer Center, Columbus, Ohio, USA; Department of Neuro-Oncology, Neurosurgery, University of Pittsburgh Medical Center, Pittsburg, Pennsylvania, USA; Department of Radiation Oncology, Thomas Jefferson University Hospital, Philadelphia, Pennsylvania, USA; Department of Neurology, Barrow Neurological Institute, Phoenix, Arizona, USA; Department of Radiation Oncology, Novant Health Forsyth Medical Center, Winston-Salem, North Carolina, USA; Department of Medicine, School of Medicine and Public Health, University of Wisconsin, Madison, Wisconsin, USA; Department of Obstetrics and Gynecology, University of Washington Medical Center, Seattle, Washington, USA; Department of Neuro-Oncology, Orlando Health Cancer Institute, Orlando, Florida, USA; Department of Radiation Oncology, University of Alabama at Birmingham Medical Center, Birmingham, Alabama, USA; Department of Radiation Oncology, Washington University, St. Louis, Missouri, USA; Department of Medical Oncology/Hematology, Sparrow HH Cancer Center, Lansing, Michigan, USA; Department of Radiation Oncology, University of Maryland Medical Systems, Baltimore, Maryland, USA; Department of Hematology, Reading Hospital, Reading, Pennsylvania, USA; Department of Radiation Oncology, Medical University of South Carolina, Charleston, South Carolina, USA; Department of Hematology Oncology, University of Cincinnati, Cincinnati, Ohio, USA; Department of Statistics, NRG Oncology Statistics and Data Management Center, Philadelphia, Pennsylvania, USA; Department of Statistics, University of Chicago, Chicago, Illinois, USA; Department of Radiation Oncology, Miami Cancer Institute, Miami, Florida, USA (M.P.M.)

**Keywords:** angiogenesis, cediranib, glioblastoma, vascular endothelial growth factor

## Abstract

**Background:**

A randomized, phase II, placebo-controlled, and blinded clinical trial (NCT01062425) was conducted to determine the efficacy of cediranib, an oral pan-vascular endothelial growth factor receptor tyrosine kinase inhibitor, versus placebo in combination with radiation and temozolomide in newly diagnosed glioblastoma.

**Methods:**

Patients with newly diagnosed glioblastoma were randomly assigned 2:1 to receive (1) cediranib (20 mg) in combination with radiation and temozolomide; (2) placebo in combination with radiation and temozolomide. The primary endpoint was 6-month progression-free survival (PFS) based on blinded, independent radiographic assessment of postcontrast T1-weighted and noncontrast T2-weighted MRI brain scans and was tested using a 1-sided *Z* test for 2 proportions. Adverse events (AEs) were evaluated per CTCAE version 4.

**Results:**

One hundred and fifty-eight patients were randomized, out of which 9 were ineligible and 12 were not evaluable for the primary endpoint, leaving 137 eligible and evaluable. 6-month PFS was 46.6% in the cediranib arm versus 24.5% in the placebo arm (*P* = .005). There was no significant difference in overall survival between the 2 arms. There was more grade ≥ 3 AEs in the cediranib arm than in the placebo arm (*P* = .02).

**Conclusions:**

This study met its primary endpoint of prolongation of 6-month PFS with cediranib in combination with radiation and temozolomide versus placebo in combination with radiation and temozolomide. There was no difference in overall survival between the 2 arms.

**Key Points**

- Cediranib is safe and feasible in newly diagnosed glioblastoma subjects.
- Cediranib improved progression-free survival (PFS) but not overall survival.
- Disease progression is challenging to define with VEGF-targeting therapies.

**Importance of the Study**

This randomized, placebo-controlled, and double-blind study confirmed the safety and benefit of an oral, pan-VEGF receptor tyrosine inhibitor, and cediranib in prolonging progression-free survival, the primary study endpoint when added to radiation and temozolomide in newly diagnosed glioblastoma. However, there was no overall survival benefit of cediranib and defining disease progression is confounded by the anti-permeability effects of a VEGF-targeting drug. These results with cediranib are consistent with those observed with bevacizumab in newly diagnosed glioblastoma.

Glioblastoma, IDH-wildtype (glioblastoma), the most common primary malignant brain tumor in adults, causes significant neurological morbidity and is associated with poor survival.^1,2^ Microvascular proliferation, a histopathological hallmark of glioblastoma, is a consequence of the high expression levels of proangiogenic cytokines, particularly of vascular endothelial growth factor (VEGF) and signaling via its endothelial tyrosine kinase receptor VEGFR2.^3–6^ Levels of VEGF and its receptor correlate with the histological grade of gliomas, with the highest levels present in glioblastoma.^7,8^Thus, novel anti-VEGF agents, such as monoclonal antibodies and tyrosine kinase inhibitors are attractive therapeutic strategies in glioblastoma.^9^ The US Food and Drug Administration approved bevacizumab, an anti-VEGF monoclonal antibody, as a monotherapy for recurrent glioblastoma in 2017 based on the radiographic response rates observed in 2 phase II trials.^10–12^ However, 2 subsequent randomized trials of bevacizumab versus placebo in combination with radiation and temozolomide demonstrated no survival benefit in newly diagnosed glioblastoma.^13,14^

Cediranib, an orally available pan-VEGFR tyrosine kinase inhibitor has a sub-nanomolar IC_50_ for VEGF receptors with additional activity against c-Kit and lower potency against PDGFRß.^15^ Based on a half-life of 22 h it can be administered once daily. In a phase II study of cediranib (45 mg/day) for patients with recurrent glioblastoma, 8/30 (27%) subjects achieved a partial radiographic response.^16,17^ Subsequently, this phase II, randomized, blinded, and placebo-controlled study (ClinicalTrials.gov identifier NCT01062425) was conducted to investigate the efficacy of cediranib, in combination with temozolomide and fractionated radiation in newly diagnosed glioblastoma.

## Patients and Methods

### Patients

Patients with newly diagnosed glioblastoma were the target population for this clinical trial. Inclusion criteria included age ≥ 18 years, pathological diagnosis of glioblastoma, and Karnofsky Performance Status (KPS) ≥ 70. Exclusion criteria included any prior anti-VEGF therapy, prior treatment with temozolomide, or prior treatment with radiation to the head or neck (except for T1 glottic cancer). All patients were required to sign an informed consent form approved by the Institutional Review Board of the enrolling institution.

The study was performed in accordance with the Declaration of Helsinki and the International Conference on Harmonization/Good Clinical Practice.

### Study Design

The study was a phase II, comparative, randomized, and multicenter trial. Patients were stratified by Recursive Partitioning Analysis (RPA), class III versus IV versus V and by the promoter methylation status of 06-methylguanine-DNA-methyltransferase (MGMT), methylated versus unmethylated versus invalid, prior to being randomized to receive cediranib (20 mg) versus placebo in combination with temozolomide (75 mg/m^2^/daily) and radiation (60 Gy in 2 Gy fractions) daily for 42 days followed by cediranib (20 mg) versus placebo in combination with temozolomide (150–200 mg/m^2^ for 5 consecutive days in 28-day cycles) for up 12 cycles maximum. Patient randomization was performed at the time of registration, with a 2:1 allocation ratio in favor of the cediranib arm, based on the permuted block design using the method described by Zelen.^18^ The primary endpoint of this study is 6-month progression-free survival (PFS) using MacDonald criteria.^19^ Secondary endpoints of this study included overall survival (OS), PFS, treatment-related toxicity, and the association between MGMT methylation status and clinical outcomes.

The sample size calculation would address whether the addition of cediranib to concurrent chemoradiation and standard temozolomide would improve the 6-month PFS rate in patients with glioblastoma. The null hypothesis was that the 6-month PFS rates for both arms were 50%, and the alternative hypothesis was that patients receiving the experimental regimen would have a 6-month PFS rate of 66%. With 150 eligible patients, there would be an 80% statistical power to detect the 16% absolute increase in 6-month PFS at a significance level of 0.15, using a 1-sided *Z* test for 2 proportions.^20^ Guarding against up to a 47% rate for patients who were retrospectively found ineligible or did not get randomized due to early disease progression, patient refusal, insufficient tissues, or other reasons, 283 patients were required to be enrolled in order to have 150 eligible and randomized patients.

Imaging reviews of disease progression by 6 months were first performed locally. The central radiology review of progression was performed by a team of reviewers including 2 readers and an adjudicator, per MacDonald criteria. The reviewers were blinded to treatment assignment and clinical information (except for neurologic function and KPS). If there was a disagreement between the 2 readers, the adjudicator provided the final determination of the progression status. Out of 34 patients judged by local sites to have disease progression by 6 months, 30 cases (88%) were confirmed by central reviews. However, among 83 patients who were judged to be progression-free by 6 months by local assessments, only 49 cases (59%) were confirmed by central reviews; the central reviews identified 34 progressors who were judged to be progression-free by 6 months per local reads.

OS was measured from the date of randomization to the date of death, or otherwise, the last follow-up date on which the patient was reported alive. PFS was measured from the date of randomization to the date of first progression, or death, or otherwise, the last follow-up date on which the patient was reported alive without progression. OS and PFS rates were estimated using the Kaplan–Meier method,^21^ and differences between the 2 treatment arms were tested using the log-rank test.^22^ Multivariate analyses for OS and PFS were performed using the Cox proportional hazards model^23^ with the stratification factors included as covariates, to assess the adjusted treatment effects. Toxicities were measured using CTCAE version 4. Differences in the observed severe toxicities (grade 3+) between the 2 arms were tested using the Chi-square test. The log-rank test was used to assess the effect of MGMT methylation status on OS and PFS, both overall (combining 2 treatment arms) and within each treatment arm. The Cox proportional hazards model was used to adjust for stratification factors (RPA class and MGMT methylation status). The proportional hazards assumption was verified using testing and graphical methods. For all the secondary endpoints, a 2-sided test with a significance level of 0.05 was used to declare statistical significance.

## Results

### Summary of Patient Enrollment

**Figure 1.**
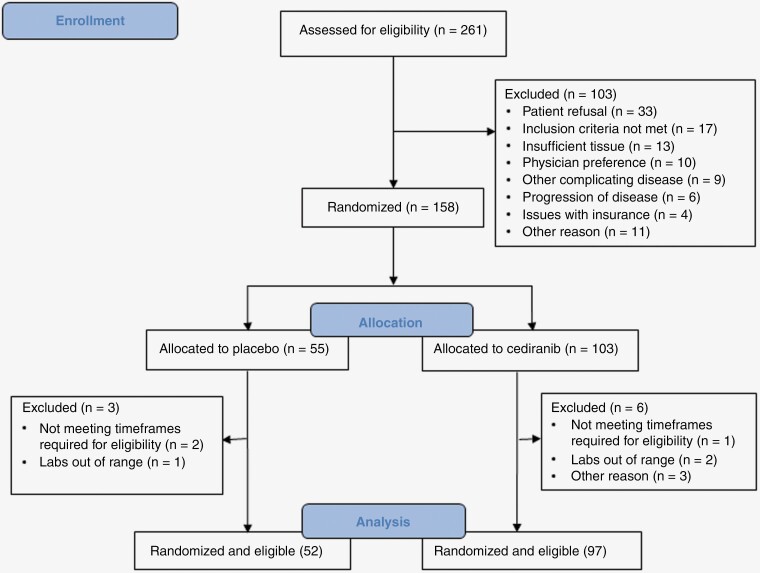
CONSORT diagram.

This study opened to accrual on February 26, 2010. Accrual was completed on May 9, 2012, with a total of 261 patients enrolled. One hundred and fifty-eight patients (60.5%) were randomized, and 9 (5.7%) of them were subsequently found ineligible. Reasons for not being randomized and ineligibility are noted in Figure 1. Therefore, for the statistical analyses, there were 52 and 97 eligible and randomized patients in the placebo and cediranib arm, respectively. Table 1 shows the distributions of pretreatment characteristics by treatment arm for all the eligible and randomized patients. The distributions of the stratification factors, MGMT methylation status, and RPA class appear balanced between the 2 treatment arms.

**Table 1.** Patient and tumor characteristics for all eligible patients in RTOG 0837

	Placebo		Cediranib		Total	
Patient or Tumor Characteristic	*n*	%	*n*	%	*n*	%
Age (years)						
Median	59		61		60	
Min–max	37–82		27–83		27–83	
Q1–Q3	51–67		54–65		53–66	
≤ 49	11	21.2	16	16.5	27	18.1
50–59	17	32.7	27	27.8	44	29.5
60–69	15	28.8	38	39.2	53	35.6
≥ 70	9	17.3	16	16.5	25	16.8
Gender						
Male	28	53.8	53	54.6	81	54.4
Female	24	46.2	44	45.4	68	45.6
Race						
Black or African American	3	5.8	4	4.1	7	4.7
White	48	92.3	93	95.9	141	94.6
Unknown or not reported	1	1.9	0	0.0	1	0.7
Ethnicity						
Hispanic or Latino	1	1.9	9	9.3	10	6.7
Not Hispanic or Latino	48	92.3	87	89.7	135	90.6
Unknown (individuals not reporting ethnicity)	3	5.8	1	1.0	4	2.7
KPS						
70–80	25	48.1	42	43.3	67	45.0
90–100	27	51.9	55	56.7	82	55.0
Surgery						
Subtotal	26	50.0	33	34.0	59	39.6
Total (gross)	24	46.2	64	66.0	88	59.1
Other	2	3.8	0	0.0	2	1.3
Neurologic Function						
No symptoms	11	21.2	29	29.9	40	26.8
Minor symptoms	26	50.0	49	50.5	75	50.3
Moderate symptoms	15	28.8	19	19.6	34	22.8
MGMT status						
Methylated	18	34.6	36	37.1	54	36.2
Unmethylated	31	59.6	57	58.8	88	59.1
Invalid	3	5.8	4	4.1	7	4.7
RPA class at randomization						
III	8	15.4	11	11.3	19	12.8
IV	34	65.4	68	70.1	102	68.5
V	10	19.2	18	18.6	28	18.8

### Treatment Adverse Events

Information on the adverse events (AEs) is presented by the treatment arm for all eligible and randomized patients who received protocol treatment. Table 2 lists the summary, overall and by system organ class, of the highest-grade AEs regardless of relationship to protocol treatment. Overall, there were 5 patients (9.6%) in the placebo arm, and 11 (12.0%) in the cediranib arm with reported grade 5 AEs. Of these 11 grade 5 AEs, 4/11 (3 infectious and 1 neurological) grade 5 events were deemed possibly, probably, or definitely related to cediranib. The proportions of patients with grade ≥ 3 AEs were summarized and compared between the treatment arms. Out of all eligible and randomized patients who received protocol treatment, 35 (67.3%) from the placebo arm and 77 (83.7%) from the cediranib arm had reported grade ≥ 3 AEs regardless of relationship to protocol treatment, resulting in a *P*-value of .02.

**Table 2.** Distribution of RTOG 0837 patients by highest grade adverse event by system organ class for all reported adverse events without regard to attribution

	Placebo (*n* = 52)					Cediranib (*n* = 92)				
System Organ Class	*n* and (%) of Patients by Grade					*n* and (%) of Patients by Grade				
	1	2	3	4	5	1	2	3	4	5
Overall highest grade	2	12	19	11	5	1	13	44	22	11
	(3.8)	(23.1)	(36.5)	(21.2)	(9.6)	(1.1)	(14.1)	(47.8)	(23.9)	(12.0)
Blood and lymphatic system ­disorders	16	2	5	2	0	19	7	7	3	0
	(30.8)	(3.8)	(9.6)	(3.8)	(0.0)	(20.7)	(7.6)	(7.6)	(3.3)	(0.0)
Cardiac disorders	2	2	0	0	0	7	1	0	1	0
	(3.8)	(3.8)	(0.0)	(0.0)	(0.0)	(7.6)	(1.1)	(0.0)	(1.1)	(0.0)
Ear and labyrinth disorders	8	2	0	0	0	7	4	0	0	0
	(15.4)	(3.8)	(0.0)	(0.0)	(0.0)	(7.6)	(4.3)	(0.0)	(0.0)	(0.0)
Endocrine disorders	2	1	0	0	0	7	3	1	0	0
	(3.8)	(1.9)	(0.0)	(0.0)	(0.0)	(7.6)	(3.3)	(1.1)	(0.0)	(0.0)
Eye disorders	6	1	0	0	0	21	1	0	0	0
	(11.5)	(1.9)	(0.0)	(0.0)	(0.0)	(22.8)	(1.1)	(0.0)	(0.0)	(0.0)
Gastrointestinal disorders	17	15	2	0	1	18	49	16	0	0
	(32.7)	(28.8)	(3.8)	(0.0)	(1.9)	(19.6)	(53.3)	(17.4)	(0.0)	(0.0)
General disorders and administration site conditions	13	25	7	0	2	16	47	16	0	2
	(25.0)	(48.1)	(13.5)	(0.0)	(3.8)	(17.4)	(51.1)	(17.4)	(0.0)	(2.2)
Hepatobiliary disorders	0	0	0	0	0	1	0	1	0	0
	(0.0)	(0.0)	(0.0)	(0.0)	(0.0)	(1.1)	(0.0)	(1.1)	(0.0)	(0.0)
Immune system disorders	0	1	0	0	0	1	1	0	0	0
	(0.0)	(1.9)	(0.0)	(0.0)	(0.0)	(1.1)	(1.1)	(0.0)	(0.0)	(0.0)
Infections and infestations	2	10	6	2	0	3	16	9	0	4
	(3.8)	(19.2)	(11.5)	(3.8)	(0.0)	(3.3)	(17.4)	(9.8)	(0.0)	(4.3)
Injury, poisoning, and procedural complications	13	2	1	0	0	18	8	1	0	0
	(25.0)	(3.8)	(1.9)	(0.0)	(0.0)	(19.6)	(8.7)	(1.1)	(0.0)	(0.0)
Investigations	8	10	10	8	0	24	24	16	21	0
	(15.4)	(19.2)	(19.2)	(15.4)	(0.0)	(26.1)	(26.1)	(17.4)	(22.8)	(0.0)
Metabolism and nutrition disorders	15	10	11	2	0	22	28	18	6	0
	(28.8)	(19.2)	(21.2)	(3.8)	(0.0)	(23.9)	(30.4)	(19.6)	(6.5)	(0.0)
Musculoskeletal and connective tissue disorders	7	3	9	0	0	15	21	11	0	0
	(13.5)	(5.8)	(17.3)	(0.0)	(0.0)	(16.3)	(22.8)	(12.0)	(0.0)	(0.0)
Neoplasms benign, malignant, and unspecified (incl cysts and polyps)	0	0	0	0	1	1	0	0	0	2
	(0.0)	(0.0)	(0.0)	(0.0)	(1.9)	(1.1)	(0.0)	(0.0)	(0.0)	(2.2)
Nervous system disorders	14	13	14	1	0	20	29	18	1	3
	(26.9)	(25.0)	(26.9)	(1.9)	(0.0)	(21.7)	(31.5)	(19.6)	(1.1)	(3.3)
Psychiatric disorders	9	12	2	1	0	17	18	2	0	0
	(17.3)	(23.1)	(3.8)	(1.9)	(0.0)	(18.5)	(19.6)	(2.2)	(0.0)	(0.0)
Renal and urinary disorders	5	5	1	0	0	15	6	1	0	0
	(9.6)	(9.6)	(1.9)	(0.0)	(0.0)	(16.3)	(6.5)	(1.1)	(0.0)	(0.0)
Reproductive system and breast disorders	2	0	0	0	0	2	1	0	0	0
	(3.8)	(0.0)	(0.0)	(0.0)	(0.0)	(2.2)	(1.1)	(0.0)	(0.0)	(0.0)
Respiratory, thoracic, and mediastinal disorders	13	1	0	0	1	24	7	5	2	0
	(25.0)	(1.9)	(0.0)	(0.0)	(1.9)	(26.1)	(7.6)	(5.4)	(2.2)	(0.0)
Skin and subcutaneous tissue disorders	19	15	1	0	0	33	27	5	0	0
	(36.5)	(28.8)	(1.9)	(0.0)	(0.0)	(35.9)	(29.3)	(5.4)	(0.0)	(0.0)
Social circumstances	0	0	0	0	0	1	0	0	0	0
	(0.0)	(0.0)	(0.0)	(0.0)	(0.0)	(1.1)	(0.0)	(0.0)	(0.0)	(0.0)
Surgical and medical procedures	0	0	0	0	0	0	1	1	0	0
	(0.0)	(0.0)	(0.0)	(0.0)	(0.0)	(0.0)	(1.1)	(1.1)	(0.0)	(0.0)
Vascular disorders	3	8	6	1	0	5	23	17	2	0
	(5.8)	(15.4)	(11.5)	(1.9)	(0.0)	(5.4)	(25.0)	(18.5)	(2.2)	(0.0)

### Test for the Primary Endpoint

The median follow-up time for all eligible patients who were still alive at the time of the analyses was 36.7 months, with a range of 2.2–53.9 months. Out of the 149 eligible and randomized patients for both arms, 137 (91.9%) were evaluable for 6-month PFS. Three patients from the placebo arm and 9 patients from the cediranib arm were not evaluable for the primary endpoint due to withdrawal before 6 months, scan not evaluable, or no protocol treatment given. Based on central radiology reviews on the primary endpoint, the 6-month PFS rate for the cediranib arm was 46.6%, as compared to 24.5% for the placebo arm, resulting in a *P*-value of .005 by 1-sided *Z* test for 2 proportions. This suggested that the experimental regimen significantly improved 6-month PFS for this patient population, as compared to the treatment of the placebo arm.

### Results for the Secondary Endpoints

**Figure 2.**
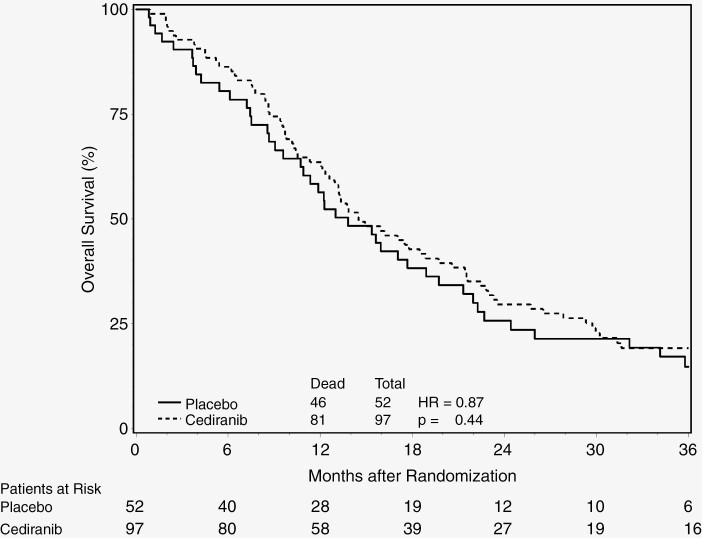
Overall survival by treatment arm.

**Figure 3.**
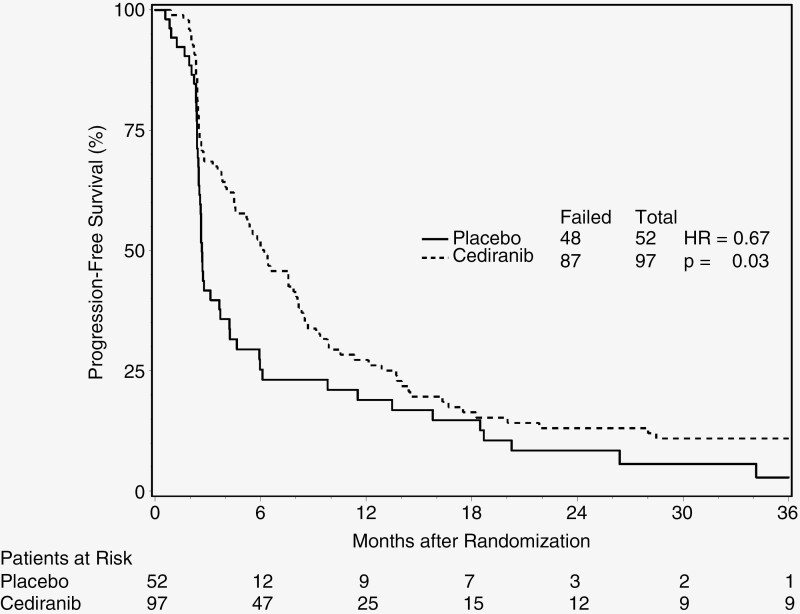
Progression-free survival by treatment arm.

The median survival time (MST) was 14.5 months (95% CI: 12.3–19.7 months) for the cediranib arm, and 13.8 months (95% CI: 9.6–18.9 months) for the placebo arm, with a hazard ratio (HR) of 0.87 (95% CI: 0.60–1.24; *P*-value = .44). The median PFS time was 6.2 months (95% CI: 4.5–8.1 months) for the cediranib arm, and 2.7 months (95% CI: 2.5–3.7 months) for the placebo arm. The corresponding HR for PFS was 0.67 (95% CI: 0.47–0.95) with a *P*-value of .03. The Kaplan–Meier curves on OS and PFS by treatment arm are demonstrated in Figures 2 and 3, respectively.

Tables 3 and 4 show the results of Cox proportional hazards models for OS and PFS. After adjusting for the stratification factors, the HR of the cediranib effect on OS was 0.91 (95% CI: 0.62–1.34) with a *P*-value of 0.65 and the HR of the cediranib effect on PFS was 0.67 (95% CI: 0.46–0.97) with a *P*-value of 0.04.

**Table 3.** Cox proportional hazards model for overall survival

Variable (Bolded value has unfavorable outcome)	*P*-value	Hazard Ratio (95% CI)
Assigned treatment (**Placebo** vs. Cediranib)	0.648	0.91 (0.62, 1.34)
MGMT (Methylated vs. **Unmethylated**)	< 0.001	2.11 (1.40, 3.17)
RPA (RPA III vs. **IV**)	0.014	2.22 (1.18, 4.20)
RPA (RPA III vs. **V**)	< 0.001	5.72 (2.74, 11.97)

**Table 4.** Cox proportional hazards model for PFS

Variable (Bolded Value has Unfavorable Outcome)	*P*-value	Hazard Ratio (95% CI)
Assigned treatment (**Placebo** vs. Cediranib)	0.036	0.67 (0.46, 0.97)
MGMT (methylated vs. **Unmethylated**)	0.950	0.99 (0.68, 1.44)
RPA (RPA III vs. **IV**)	0.458	1.23 (0.71, 2.13)
RPA (RPA III vs. **V**)	0.010	2.39 (1.23, 4.63)

The 6-month PFS rates were also compared between the treatment arms by gender. For males, the 6-month PFS rates for the cediranib and placebo arm were 55.3% (95% CI: 40.1–69.8%) and 26.9% (95% CI: 11.6–47.8%), respectively. For females, the 6-month PFS rates for the cediranib and placebo arm were 36.6% (95% CI: 22.1–53.1%) and 21.7% (95% CI: 7.5–43.7%), respectively.

### Results of the Analyses on the MGMT Methylation Status

OS and PFS rates by MGMT methylation status were assessed for all the eligible and randomized patients from both arms. The MST was 13.0 months (95% CI: 10.9–15.4 months) for unmethylated patients, and 29.3 months (95% CI: 17.1–36.2 months) for methylated patients, with a *P*-value < .001 and the HR was 2.13 (95% CI: 1.44–3.15). The median PFS time was 5.2 months (95% CI: 3.8–6.4 months) for unmethylated patients, and 3.2 months (95% CI: 2.5–8.0 months) for methylated patients, with a *P*-value of.52 and an HR of 1.13 (95% CI: 0.78–1.63). The Kaplan–Meier curves on OS and PFS by MGMT methylation status for patients from both arms are demonstrated in Supplementary Figures S1 and S2, respectively online only.

OS and PFS rates by MGMT methylation status were assessed for patients in the placebo arm. The MST was 13.4 months (95% CI: 10.7–19.7 months) for unmethylated patients, and 17.7 months (95% CI: 2.4–39.6 months) for methylated patients, with a *P*-value = .20 and an HR of 1.56 (95% CI: 0.79–3.06). The median PFS time was 3.2 months (95% CI: 2.6–6.1 months) for unmethylated patients, and 2.4 months (95% CI: 2.1–2.7 months) for methylated patients, with a *P*-value = .054, and an HR of 0.55 (95% CI: 0.30–1.02).

Similar analyses were performed for patients in the cediranib arm. The MST was 13.0 months (95% CI: 10.3–14.5 months) for unmethylated patients, and 30.0 months (95% CI: 18.9–40.1 months) for methylated patients, with an HR of 2.59 (95% CI: 1.58–4.25; *P*-value < .001). The median PFS time was 6.0 months (95% CI: 4.1–7.9 months) for unmethylated patients, and 6.2 months (95% CI: 2.5–16.3 months) for methylated patients, with an HR of 1.51 (95% CI: 0.96–2.40; *P*-value = .07). As an exploratory analysis, we examined the prognostic value of MGMT methylation status for patients in the cediranib arm, adjusting for RPA class in the Cox proportional hazards model: the HR for MGMT unmethylated versus methylated for OS and PFS were 2.91 (95% CI: 1.74–4.86) and 1.46 (95% CI: 0.91–2.35), respectively.

## Discussion

Anti-angiogenic therapy is a beneficial component in the treatment of multiple solid tumors given the importance of adequate blood supply for tumor growth and metastasis.^3^ Despite promising preclinical data and early clinical trials, anti-angiogenic agents have failed to show an overall survival benefit in randomized controlled trials of patients with glioblastoma.^13,14,24,25^ In particular, agents targeting VEGF appear to prolong PFS, possibly improve quality of life in some patients, and decrease steroid usage, yet the trials to date have demonstrated no improvement in overall survival. Moreover, it remains unclear whether the extension of “progression”-free survival observed in multiple randomized trials of anti-VEGF therapies represents a delay of tumor growth versus an artefactual effect of this class of agents. The anti-permeability effects of VEGF inhibitors reduce contrast extravasation through tumor blood vessels complicating the interpretation of postcontrast CT and MRI studies.

The addition of cediranib to standard concurrent chemoradiation and adjuvant temozolomide in this trial significantly improved the 6-month PFS rate in patients with newly diagnosed glioblastoma. However, OS was not significantly improved, and the rate of severe toxicities was significantly increased.

The prognostic value of MGMT methylation status for patients in the cediranib arm reflected a similar pattern, implying no significant biologic effect.

Although the primary endpoint of NRG Oncology/RTOG 0837, PFS at 6 months, was met, the anti-permeability effects of cediranib may confound the interpretation of postcontrast brain CT and MRI scans and raise questions about the validity of this endpoint in assessing the utility of VEGF inhibitors. However, no alternative imaging endpoint has been validated as a method of identifying tumor progression in this setting. Development of cediranib is ongoing in glioblastoma and other solid tumors, including ovarian carcinoma. Preclinical models demonstrate that the hypoxia that results from inhibition of angiogenesis disrupts homologous recombination repair mechanisms rendering cancer cells susceptible to inhibition of poly (ADP-ribose) polymerase (PARP) even in the absence of BRCA 1/2 mutations.^26,27^ Cediranib in combination with PARP inhibition is being studied in a recurrent glioblastoma trial (NCT02974621).

The results of NRG Oncology/RTOG 0837 are consistent with the results observed in 4 randomized, controlled trials of bevacizumab or cediranib in newly diagnosed and recurrent glioblastoma.^13,14,24,25^ The lack of an overall survival benefit with VEGF inhibitors with different mechanisms of action (VEGF-A ligand sequestration, VEGF receptor tyrosine kinase receptor inhibition) in both the newly diagnosed and recurrent disease settings raises the question of whether these drugs have any role as single agents in the treatment of glioblastoma patients. While the confounding impact of patient crossover from the placebo arms to ultimately receive anti-VEGF therapy exists for all these studies the precise impact of such crossover on overall survival cannot be defined. Moreover, there are no predictive markers to identify glioblastoma subpopulations most likely to benefit from anti-VEGF therapy. While the anti-permeability effects of VEGF inhibitors confound the interpretation of postcontrast brain imaging studies this biological effect has the benefit of reducing brain edema as demonstrated by the steroid-sparing impact of these agents. While neuropsychological and quality of life outcomes are conflicting in these prior studies there is at least some indication of benefit. Future studies are needed to identify predictive biospecimen or imaging markers of glioblastoma subpopulations most likely to benefit from anti-VEGF therapies and to identify the molecular mechanisms involved in the development of resistance to these drugs. The latter will inform future clinical trials of combinations of anti-VEGF agents with inhibitors of other proangiogenic signal transduction pathways.

## Supplementary Material

vdad116_suppl_Supplementary_Figures_1_2

vdad116_suppl_Supplementary_Figures_S1

vdad116_suppl_Supplementary_Figures_S2

## References

[1] WHO Classification of Tumours Editorial Board. Central Nervous System Tumours. Lyon (France): International Agency for Research on Cancer; 2021 (WHO classification of tumours series, 5^th^ ed.; vol. 6)

[2] Stupp R , Mason WP, van den Bent MJ, et al; European Organisation for Research and Treatment of Cancer Brain Tumor and Radiotherapy Groups. Radiotherapy plus concomitant and adjuvant temozolomide for glioblastoma. N Engl J Med. 2005;352(10):987–996. 15758009 10.1056/NEJMoa043330

[3] Jain RK , di Tomaso E, Duda DG, et al. Angiogenesis in brain tumours. Nat Rev Neurosci. 2007;8(8):610–622. 17643088 10.1038/nrn2175

[4] Holash J , Maisonpierre PC, Compton D, et al. Vessel cooption, regression, and growth in tumors mediated by angiopoietins and VEGF. Science. 1999;284(5422):1994–1998. 10373119 10.1126/science.284.5422.1994

[5] Shweiki D , Itin A, Soffer D, Keshet E. Vascular endothelial growth factor induced by hypoxia may mediate hypoxia-initiated angiogenesis. Nature. 1992;359(6398):843–845. 1279431 10.1038/359843a0

[6] Millauer B , Shawver LK, Plate KH, Risau W, Ullrich A. Glioblastoma growth inhibited in vivo by a dominant-negative Flk-1 mutant. Nature. 1994;367(6463):576–579. 8107827 10.1038/367576a0

[7] Samoto K , Ikezaki K, Ono M, et al. Expression of vascular endothelial growth factor and its possible relation with neovascularization in human brain tumors. Cancer Res. 1995;55(5):1189–1193. 7532545

[8] Schmidt NO , Westphal M, Hagel C, et al. Levels of vascular endothelial growth factor, hepatocyte growth factor/scatter factor and basic fibroblast growth factor in human gliomas and their relation to angiogenesis. Int J Cancer. 1999;84(1):10–18. 9988225 10.1002/(sici)1097-0215(19990219)84:1<10::aid-ijc3>3.0.co;2-l

[9] Jain RK , Duda DG, Clark JW, Loeffler JS. Lessons from phase III clinical trials on anti-VEGF therapy for cancer. Nat Clin Pract Oncol. 2006;3(1):24–40. 16407877 10.1038/ncponc0403

[10] Friedman HS , Prados MD, Wen PY, et al. Bevacizumab alone and in combination with irinotecan in recurrent glioblastoma. J Clin Oncol. 2009;27(28):4733–4740. 19720927 10.1200/JCO.2008.19.8721

[11] Kreisl TN , Kim L, Moore K, et al. Phase II trial of single-agent bevacizumab followed by bevacizumab plus irinotecan at tumor progression in recurrent glioblastoma. J Clin Oncol. 2009;27(5):740–745. 19114704 10.1200/JCO.2008.16.3055 PMC2645088

[12] Cohen MH , Shen YL, Keegan P, Pazdur R. FDA drug approval summary: bevacizumab (Avastin) as treatment of recurrent glioblastoma multiforme. Oncologist. 2009;14(11):1131–1138. 19897538 10.1634/theoncologist.2009-0121

[13] Chinot OL , Wick W, Mason W, et al. Bevacizumab plus radiotherapy-temozolomide for newly diagnosed glioblastoma. N Engl J Med. 2014;370(8):709–722. 24552318 10.1056/NEJMoa1308345

[14] Gilbert MR , Dignam JJ, Armstrong TS, et al. A randomized trial of bevacizumab for newly diagnosed glioblastoma. N Engl J Med. 2014;370(8):699–708. 24552317 10.1056/NEJMoa1308573 PMC4201043

[15] Batchelor TT , Sorensen AG, di Tomaso E, et al. AZD2171, a pan-VEGF receptor tyrosine kinase inhibitor, normalizes tumor vasculature and alleviates edema in glioblastoma patients. Cancer Cell. 2007;11(1):83–95. 17222792 10.1016/j.ccr.2006.11.021 PMC2748664

[16] Batchelor TT , Duda DG, di Tomaso E, et al. Phase II study of cediranib, an oral pan-vascular endothelial growth factor receptor tyrosine kinase inhibitor, in patients with recurrent glioblastoma. J Clin Oncol. 2010;28(17):2817–2823. 20458050 10.1200/JCO.2009.26.3988 PMC2903316

[17] Wen PY , Macdonald DR, Reardon DA, et al. Updated response assessment criteria for high-grade gliomas: response assessment in neuro-oncology working group. J Clin Oncol. 2010;28(11):1963–1972. 20231676 10.1200/JCO.2009.26.3541

[18] Zelen M. The randomization and stratification of patients to clinical trials. J Chronic Dis. 1974;27(7-8):365–375. 4612056 10.1016/0021-9681(74)90015-0

[19] Macdonald DR , Cascino TL, Schold SC, Jr, Cairncross JG. Response criteria for phase II studies of supratentorial malignant glioma. J Clin Oncol. 1990;8(7):1277–1280. 2358840 10.1200/JCO.1990.8.7.1277

[20] Ott L. An Introduction to Statistical Methods and Data Analysis. Belmont, Calif.: Duxbury Press; 1993.

[21] Kaplan EL , Meier P. Nonparametric estimation from incomplete observations. J Am Stat Assoc. 1958;53:457–481.

[22] Mantel N. Evaluation of survival data and two new rank order statistics arising in its consideration. Cancer Chemother Rep. 1966;50(3):163–170. 5910392

[23] Cox DR. Regression models and life-tables. J R Stat Soc Series B 1972;34:187–202.

[24] Wick W , Gorlia T, Bendszus M, et al. Lomustine and Bevacizumab in progressive glioblastoma. N Engl J Med. 2017;377(20):1954–1963. 29141164 10.1056/NEJMoa1707358

[25] Batchelor TT , Mulholland P, Neyns B, et al. Phase III randomized trial comparing the efficacy of cediranib as monotherapy, and in combination with lomustine, versus lomustine alone in patients with recurrent glioblastoma. J Clin Oncol. 2013;31(26):3212–3218. 23940216 10.1200/JCO.2012.47.2464 PMC4021043

[26] Klein TJ , Glazer PM. The tumor microenvironment and DNA repair. Semin Radiat Oncol. 2010;20(4):282–287. 20832021 10.1016/j.semradonc.2010.05.006 PMC2948843

[27] Lim JJ , Yang K, Taylor-Harding B, Wiedemeyer WR, Buckanovich RJ. VEGFR3 inhibition chemosensitizes ovarian cancer stemlike cells through down-regulation of BRCA1 and BRCA2. Neoplasia. 2014;16(4):343–53.e1. 24862760 10.1016/j.neo.2014.04.003 PMC4094836

