# Supplementary figures and images for: NRG/RTOG 0837: Randomized, phase II, double-blind, placebo-controlled trial of chemoradiation with or without cediranib in newly diagnosed glioblastoma
Source: Neurooncol Adv. 2023 Oct 11;5(1):vdad116. doi: 10.1093/noajnl/vdad116 (PMC10660192; doi:10.1093/noajnl/vdad116)

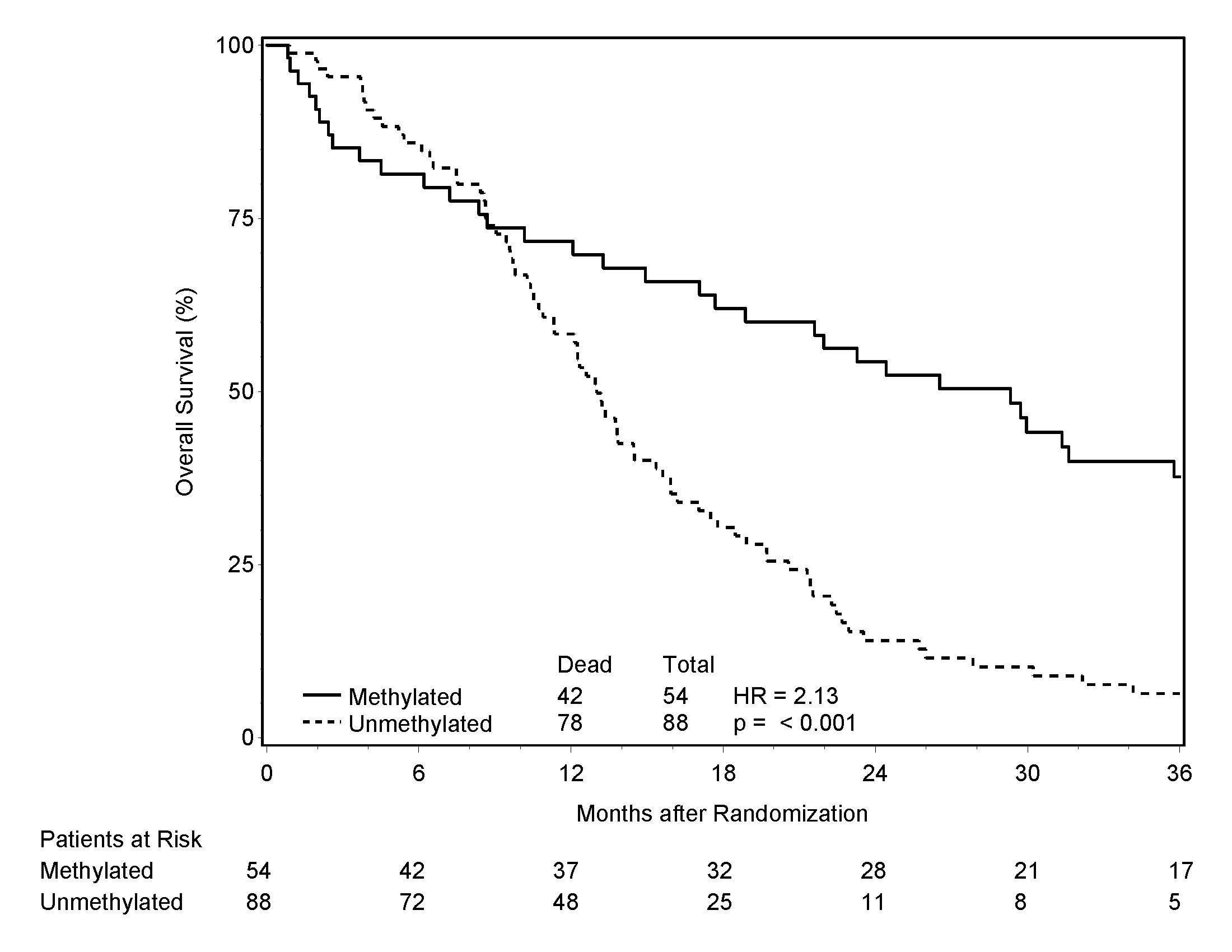

Supplement: vdad116_suppl_Supplementary_Figures_S1 [file vdad116_suppl_supplementary_figures_s1.jpeg]

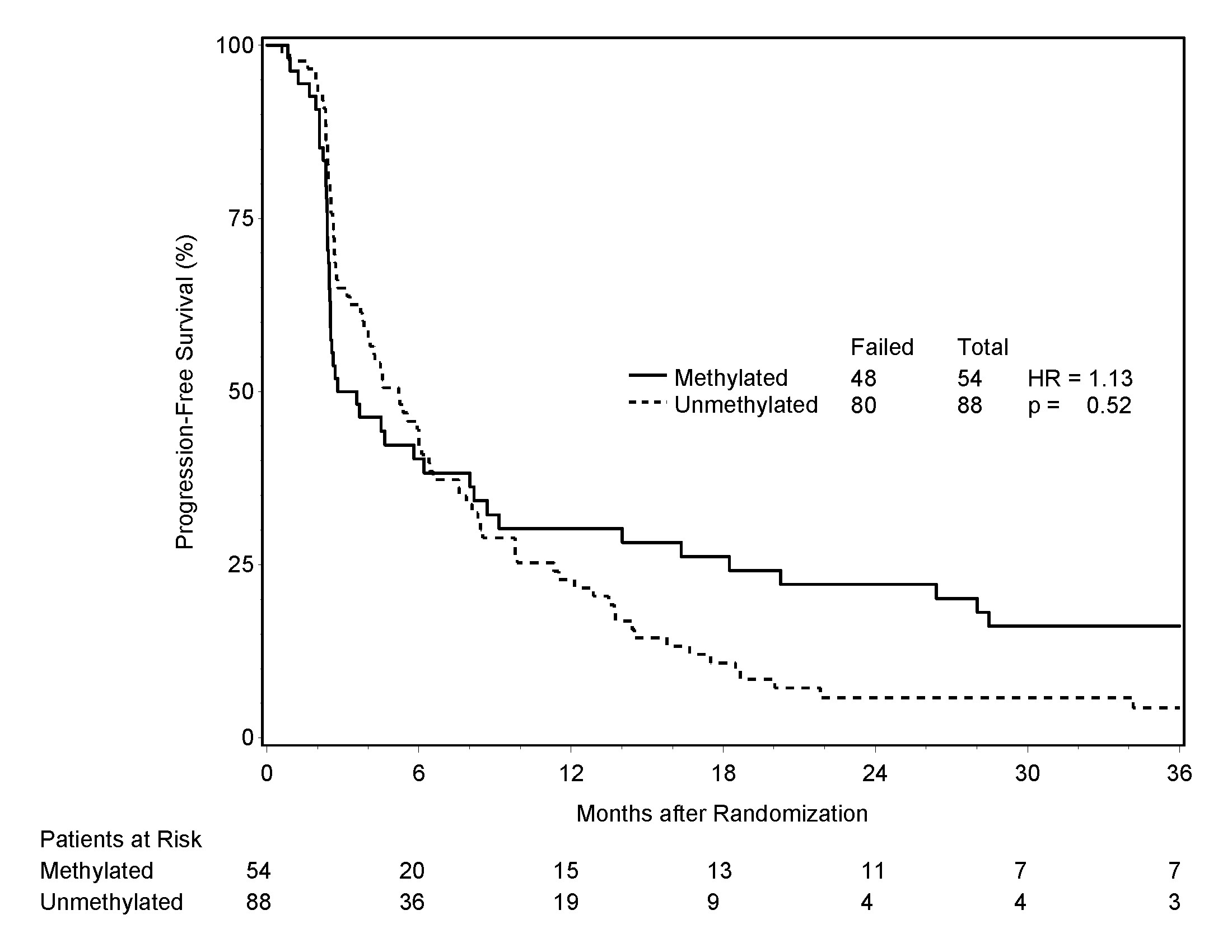

Supplement: vdad116_suppl_Supplementary_Figures_S2 [file vdad116_suppl_supplementary_figures_s2.jpeg]
